# Exploring the Efficacy of Pooled Stools in Fecal Microbiota Transplantation for Microbiota-Associated Chronic Diseases

**DOI:** 10.1371/journal.pone.0163956

**Published:** 2017-01-09

**Authors:** Abbas Kazerouni, Lawrence M. Wein

**Affiliations:** 1 Department of Electrical Engineering, Stanford University, Stanford, California, Unites States of America; 2 Graduate School of Business, Stanford University, Stanford, California, Unites States of America; Case Western Reserve University, UNITED STATES

## Abstract

Fecal microbiota transplantation is being assessed as a treatment for chronic microbiota-related diseases such as ulcerative colitis. Results from an initial randomized trial suggest that remission rates depend on unobservable features of the fecal donors and observable features of the patients. We use mathematical modeling to assess the efficacy of pooling stools from different donors during multiple rounds of treatment. In the model, there are two types of patients and two types of donors, where the patient type is observable and the donor type (effective or not effective) is not observable. In the model, clinical outcomes from earlier rounds of treatment are used to estimate the current likelihood that each donor is effective, and then each patient in each round is treated by a pool of donors that are currently deemed to be the most effective. Relative to the no-pooling case, pools of size two or three significantly increase the proportion of patients in remission during the first several rounds of treatment. Although based on data from a single randomized trial, our modeling suggests that pooling of stools – via daily cycling of encapsulated stool from several different donors – may be beneficial in fecal microbiota transplantation for chronic microbiota-related diseases.

## Introduction

Fecal microbiota transplantation (FMT), i.e., stool transplanted from a healthy donor that reconstitutes the normal microbiota community in the gut, is an effective treatment for *Clostridium difficile* infection, achieving a 90% cure rate in recurrent cases [1]. This treatment modality is now being considered for chronic and difficult-to-treat microbiota-associated diseases such as ulcerative colitis (UC). Although results from a placebo-controlled randomized trial of FMT for UC [2] show that FMT is only moderately effective (24% remission probability vs. 5% for placebo), they also reveal two interesting phenomena: remission probabilities are highly dependent on the fecal donor (one of five donors achieved seven of the nine remissions) and on the length of time that a patient has had UC (three of four patients with UC for <1 year experienced remission). While this patient heterogeneity is observable prior to treatment (i.e., via the time with UC), heterogeneity in the efficacy of donor material is not observable prior to treatment under the current state of scientific knowledge; in particular, it is not currently possible to predict whether a donor’s stools will lead to remission based on an analysis of 16s rRNA data or a more general metagenomic analysis.

This unobservable heterogeneity suggests that remission rates might be improved by pooling several different donors’ stools into each patient; this could be achieved via an encapsulated stool protocol [3] with daily cycling of pills; e.g., if the pool size is two, pills from one donor are given on odd-numbered days and pills from a second donor are administered on even-numbered days. We use mathematical modeling and analysis to propose and assess an adaptive donor allocation policy that statistically infers which donors are effective and then allocates stools (in pool sizes up to five) from the best donors to each patient in each round of treatment.

## Methods

We describe the mathematical model and then propose an algorithm that dynamically assigns patients to donor pools—or equivalently, allocates donor pools to patients—in a multi-round setting.

### The Model

We consider two versions of the model, and begin with the aspects of the model that are common to both versions. There are *N*_1_ patients who have had UC for <1 year and *N*_2_ patients who have had UC for >1 year; they are referred to as type 1 and type 2 patients. We consider *T* rounds of treatment indexed by *t* = 1, …, *T*, and let *D*_*t*_ fecal donors be available in treatment round *t*; as explained later, new donors can be added during each round before allocating treatments. Each donor is either effective (referred to as type 1) or ineffective (type 0). The probability that a donor is effective is *p*, although a donor’s type is not observable and needs to be estimated from previous treatment results. Treatment results are binary, where patients are either in remission or not in remission after receiving treatment in any given round.

In each round, each patient receives 14 days of treatment from a particular donor pool. We assume that treatment is persistent; i.e., the treatment outcome from a particular patient-donor pool pair is the same in all rounds of treatment. In particular, if a patient is in remission after treatment from a particular donor pool, then he remains in remission for as long as he receives treatment from this donor pool. Consequently, we assume that if a patient is in remission after treatment in round *t* from a particular donor pool, then that patient continues to receive treatment from this donor pool in rounds *t* + 1, …, *T*. Moreover, because a patient who is not in remission after treatment from a particular donor pool would not achieve remission for as long as treatment is continued with this donor pool, all patients who are not in remission after treatment in round *t* are reassigned to a different donor pool in round *t* + 1. Therefore, the proportion of patients in remission at the end of round *t* is nondecreasing in *t*.

Turning to the effect of pooled donors, because it takes up to 14 days of treatment to observe whether remission is achieved, we assume that daily cycling of pills from several donors (e.g., ABABAB⋯ if there are two donors and ABCABC⋯ if there are three donors, where A, B and C represent different donors) achieves the same outcome as a pooled stool from these donors. While this assumption is likely to hold for small pool sizes (e.g., two or three), the point at which this assumption breaks down has yet to be assessed empirically. In other words, we implicitly assume that—regardless of pool size (although we only consider pools up to size five)—the probability of engraftment of specific microbes is not lowered due to pooling, and that once the microbes are engrafted, the actual equilibrium and associated clinical outcome would be the same. This assumption is consistent with Lotka-Volterra models of intestinal microbiota [4]-[6], where—after a perturbation due to FMT—the new microbiota equilibrium in the patient will be independent of the initial quantities of microbes from the donor pool beyond these microbes’ absence or presence. While it could take longer to attain the new equilibrium if the initial concentration of certain microbes is lower (i.e., due to pooling), the actual equilibrium—and hence clinical outcome—should be the same.

Due to the paucity of data about FMT treatment for UC (and, in particular, the absence of data from pooled stools or from multi-round treatments), we consider two versions of the model that differ in their probabilistic assumptions about treatment outcomes, both within a pool of several donors and across rounds of treatment. In the optimistic (in the sense of achieving remission in more patients) scenario referred to as the independence version of the model, we assume that the treatment results from different donors for a particular patient are independent within a pool and independent across rounds. In the pessimistic scenario referred to as the dependence version of the model, we assume that once a patient receives a treatment outcome from a particular donor type, he experiences that same treatment outcome if he receives treatment from another donor of the same type, whether this other donor is in the same pool as the original donor (i.e., in the same round of treatment) or is in a subsequent round of treatment. Another assumption in the dependence version of the model is that the remissions achieved by type 0 donors are a subset of (i.e., subsumed by) the remissions achieved by type 1 donors; i.e., an ineffective donor is incapable of achieving remission in a patient that did not achieve remission from an effective donor, and hence a patient’s overall remission probability is independent of the number of previous times that he was unsuccessfully treated by an ineffective donor.

One way to think about these two versions of the model is that each effective donor possesses an effective unobservable factor (e.g., a microorganism) that achieves a (relatively) high remission probability, and each ineffective donor possesses an ineffective factor that achieves a lower remission probability. In the independence version, there are many of these factors and each donor possesses a different factor (be it effective or ineffective), and the patient is lacking all of these (effective and ineffective) factors; in the dependence version, each effective donor possesses the same effective factor, each ineffective donor possesses the same ineffective factor, each patient is lacking both the effective factor and the ineffective factor, and the effective factor subsumes the ineffective factor.

We now formulate and analyze the two versions of the model, culminating in the calculation of the posterior marginal probabilities that each donor is effective at the end of each round of treatment. We then present an allocation strategy that applies to both versions of the model.

### The Independence Version of the Model

In the absence of donor pooling, the remission probability when a donor of type *i* first treats a patient of type *j* is *r*_*ij*_ for *i* = 0, 1 and *j* = 1, 2, regardless of when (i.e., which round) the treatment occurs. Hence, a patient can fail treatment from a donor in round *t* and then achieve remission from a different donor of the same type in a later round. If a type *j* donor, in round *t*, is treated by a pool comprised of a mix of $s_{0}^{t}$ ineffective donors and $s_{1}^{t}$ effective donors who have never treated this patient before, then the probability of remission is $1-{\left(1-r_{0j}\right)}^{s_{0}^{t}}{\left(1-r_{1j}\right)}^{s_{1}^{t}}$. Thus, the probability of remission after *t* rounds of treatment for a patient of type *j* is

$$\begin{array}{c} 1-\prod_{\tau =1}^{t}\left[{\left(1-r_{0j}\right)}^{s_{0}^{\tau }}{\left(1-r_{1j}\right)}^{s_{1}^{\tau }}\right]. \end{array}$$ (1)

The key to the analysis is to compute the posterior marginal probability that donor *d* is of type 1 (i.e., effective) conditioned on all of the treatment results in the first *t* rounds, which is denoted by *ϵ*_*d*_(*t*). To compute this posterior probability, we need to introduce additional notation. Let *s* be the pool size (i.e., the number of donors in a pool), let $K_{t}=\left(\begin{array}{c} D_{t}\\ s \end{array}\right)$ be the number of pools of size *s* that can be formed from the *D*_*t*_ donors available in round *t*, index these pools by *k* = 1, …, *K*_*t*_, and let *A*_*k*_ be the set of *s* donors in pool *k*. For each patient *l*, let $U_{l}^{t}$ be the set of all donors from which he has received unsuccessful treatment by the end of round *t* and $V_{l}^{t}$ be the set of donors who achieved remission in patient *l* by the end of round *t*. Note that $V_{l}^{t}$ is either empty or contains the donors in the pool that achieved remission in patient *l*. Furthermore, $V_{l}^{t}\cap U_{l}^{t}$ is empty for all values of *t* and *l* because a patient is never reassigned to a donor from whom he previously received unsuccessful treatment. For *d* = 1, …, *D*_*t*_, let donor *d*’s type *E*_*d*_ equal *i* if donor *d* is of type *i*, for *i* = 0, 1. Given the donors’ types *E*_1_ = *e*_1_, *E*_2_ = *e*_2_, …, *E*_*D*_*t*__ = *e*_*D*_*t*__, define

$$\begin{array}{c} \alpha_{l}^{t}\left(\text{e}\right)=\left|\left\{d\in U_{l}^{t}:e_{d}=1\right\}\right|, \end{array}$$ (2)

$$\begin{array}{c} \beta_{l}^{t}\left(\text{e}\right)=\left|\left\{d\in U_{l}^{t}:e_{d}=0\right\}\right|, \end{array}$$ (3)

$$\begin{array}{c} \mu_{l}^{t}\left(\text{e}\right)=\left|\left\{d\in V_{l}^{t}:e_{d}=1\right\}\right|, \end{array}$$ (4)

and

$$\begin{array}{c} \nu_{l}^{t}\left(\text{e}\right)=\left|\left\{d\in V_{l}^{t}:e_{d}=0\right\}\right|, \end{array}$$ (5)

where **e** = (*e*_1_, *e*_2_, …, *e*_*D*_*t*__). In words, $\alpha_{l}^{t}$ and $\beta_{l}^{t}$ are the number of effective and ineffective donors, respectively, who have unsuccessfully treated patient *l* by the end of round *t*, and $\mu_{l}^{t}$ and $\nu_{l}^{t}$ are the number of effective and ineffective donors, respectively, who have achieved remission in patient *l* by the end of round *t*. Note that $\mu_{l}^{t}+\nu_{l}^{t}=s$ or 0, depending upon whether or not patient *l* is in remission at the end of round *t*.

The key to the analysis is the computation of the posterior joint distribution, $P(\text{E}=\text{e}|H_{t})$, of each donor’s type at the end of each round, given all previous treatment results, $H_{t}$, where **E** = (*E*_1_, *E*_2_, …, *E*_*D*_*t*__). The posterior distribution at the end of each round can be updated using only the sufficient statistics $\alpha_{l}^{t},\beta_{l}^{t},\mu_{l}^{t}$ and $\nu_{l}^{t}$ in Eqs (2)–(5). For any integer *N*, define [*N*] = {1, 2, …, *N*}. Then for $\text{e}\in {\left\{0,1\right\}}^{D_{t}}$, we have

$$\begin{array}{ccc} P_{t}\left(\text{e}\right) & = & P[\text{E}=\text{e}|H_{t}]\\ & = & \frac{1}{Z}\prod_{d=1}^{D_{t}}p^{e_{d}}{\left(1-p\right)}^{1-e_{d}}\prod_{j=1}^{2}\prod_{l\in [N_{j}]}{\left(1-r_{0j}\right)}^{\beta_{l}^{t}\left(\text{e}\right)}{\left(1-r_{1j}\right)}^{\alpha_{l}^{t}\left(\text{e}\right)}\\ & & \times (1-{\left(1-r_{0j}\right)}^{\nu_{l}^{t}\left(\text{e}\right)}{\left(1-r_{1j}\right)}^{\mu_{l}^{t}\left(\text{e}\right)}), \end{array}$$ (6)

where *Z* is the normalization constant defined as

$$\begin{array}{c} Z=\sum_{\text{e}\in {\left\{0,1\right\}}^{D_{t}}}\prod_{d=1}^{D_{t}}p^{e_{d}}{\left(1-p\right)}^{1-e_{d}}\prod_{j=1}^{2}\prod_{l\in [N_{j}]}{\left(1-r_{0j}\right)}^{\beta_{l}^{t}\left(\text{e}\right)}{\left(1-r_{1j}\right)}^{\alpha_{l}^{t}\left(\text{e}\right)}\left(1-{\left(1-r_{0j}\right)}^{\nu_{l}^{t}\left(\text{e}\right)}{\left(1-r_{1j}\right)}^{\mu_{l}^{t}\left(\text{e}\right)}\right). \end{array}$$ (7)

A key observation is that the updated posterior probability at the end of the previous round together with the treatment outcomes observed in the current round are sufficient to update the posterior probability at the end of the current round. Specifically, defining $a_{l}^{t}\left(\text{e}\right)=\alpha_{l}^{t}\left(\text{e}\right)-\alpha_{l}^{t-1}\left(\text{e}\right),b_{l}^{t}\left(\text{e}\right)=\beta_{l}^{t}\left(\text{e}\right)-\beta_{l}^{t-1}\left(\text{e}\right),m_{l}^{t}\left(\text{e}\right)=\mu_{l}^{t}\left(\text{e}\right)-\mu_{l}^{t-1}\left(\text{e}\right)$ and $n_{l}^{t}\left(\text{e}\right)=\nu_{l}^{t}\left(\text{e}\right)-\nu_{l}^{t-1}\left(\text{e}\right)$ (which are all observable after treatment in round *t*), and taking

$$\begin{array}{c} P_{0}\left(\text{e}\right)=\frac{\prod_{d=1}^{D_{1}}p^{e_{d}}{\left(1-p\right)}^{1-e_{d}}}{\sum_{{\text{e}}^{\prime }\in {\left\{0,1\right\}}^{D_{1}}}\prod_{d=1}^{D_{1}}p^{e_{d}^{\prime }}{\left(1-p\right)}^{1-e_{d}^{\prime }}}, \end{array}$$ (8)

we have, for any *t* = 1, 2, …, *T*,

$$\begin{array}{c} P_{t}\left(\text{e}\right)=\frac{1}{Z}P_{t-1}\left(\text{e}\right)\prod_{j=1}^{2}\prod_{l\in [N_{j}]}{\left(1-r_{0j}\right)}^{b_{l}^{t}\left(\text{e}\right)}{\left(1-r_{1j}\right)}^{a_{l}^{t}\left(\text{e}\right)}\left(1-{\left(1-r_{0j}\right)}^{n_{l}^{t}\left(\text{e}\right)}{\left(1-r_{1j}\right)}^{m_{l}^{t}\left(\text{e}\right)}\right), \end{array}$$ (9)

where the normalization constant *Z* is

$$\begin{array}{c} Z=\sum_{\text{e}\in {\left\{0,1\right\}}^{D_{t}}}P_{t-1}\left(\text{e}\right)\prod_{j=1}^{2}\prod_{l\in [N_{j}]}{\left(1-r_{0j}\right)}^{b_{l}^{t}\left(\text{e}\right)}{\left(1-r_{1j}\right)}^{a_{l}^{t}\left(\text{e}\right)}\left(1-{\left(1-r_{0j}\right)}^{n_{l}^{t}\left(\text{e}\right)}{\left(1-r_{1j}\right)}^{m_{l}^{t}\left(\text{e}\right)}\right). \end{array}$$ (10)

Given the posterior joint probability of donors types in Eqs (9) and (10), the posterior marginal probability of each donor *d* being of type 1 at the end of round *t* is

$$\begin{array}{ccc} \epsilon_{d}\left(t\right) & = & P[E_{d}=1|H_{t}],\\ & = & \sum_{\text{e}:e_{d}=1}P_{t}\left(\text{e}\right). \end{array}$$ (11)

### The Dependence Version of the Model

In the dependence version, we assume perfect temporal dependence in the treatment outcome. More specifically, if a type *j* patient is treated by a pool with at least one effective donor in round 1, then remission is achieved with probability *r*_1*j*_. But if that patient does not achieve remission, then the patient never achieves remission in any future rounds, regardless of treatment. If a patient is treated by a pool with all ineffective donors in round 1, then remission is achieved with probability *r*_0*j*_. If remission is not achieved, then the patient never achieves remission in subsequent rounds when treated by a pool with all ineffective donors, and if the patient is subsequently treated by a pool with at least one effective donor, then remission is achieved with probability $\frac{r_{1j}-r_{0j}}{1-r_{0j}}$, so that the patient’s overall remission probability is also *r*_1*j*_.

**Fig 1:**
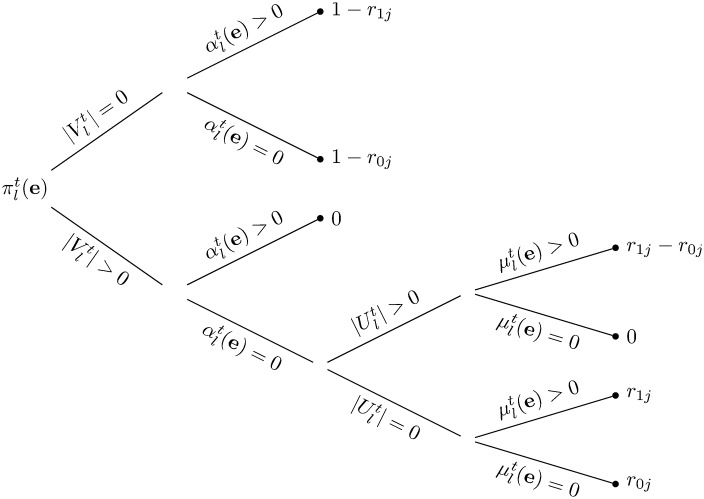
A graphical depiction of Eq (12).

The first step in deriving the posterior marginal probability, *ϵ*_*d*_(*t*), that donor *d* is of type 1 at the end of round *t*, is to compute the likelihood $\pi_{l}^{t}\left(\text{e}\right)$ of the data observed for patient *l* of type *j* through the end of round *t*, conditioned on the vector **e** of donor states. This likelihood, which is graphically depicted in Fig 1, is

$$\begin{array}{c} \pi_{l}^{t}\left(\text{e}\right)=\left\{ \begin{array}{cc} 1-r_{1j} & \text{if}\;\left|V_{l}^{t}\right|=0,\;\alpha_{l}^{t}\left(\text{e}\right)>0;\\ 1-r_{0j} & \text{if}\;\left|V_{l}^{t}\right|=0,\;\alpha_{l}^{t}\left(\text{e}\right)=0;\\ 0 & \text{if}\;|V_{l}^{t}|>0,\;\alpha_{l}^{t}\left(\text{e}\right)>0;\\ 0 & \text{if}\;|V_{l}^{t}|>0,\;\alpha_{l}^{t}\left(\text{e}\right)=0,\;\left|U_{l}^{t}\right|>0,\;\mu_{l}^{t}\left(\text{e}\right)=0;\\ r_{1j}-r_{0j} & \text{if}\;|V_{l}^{t}|>0,\;\alpha_{l}^{t}\left(\text{e}\right)=0,\;\left|U_{l}^{t}\right|>0,\;\mu_{l}^{t}\left(\text{e}\right)>0;\\ r_{1j} & \text{if}\;|V_{l}^{t}|>0,\;\alpha_{l}^{t}\left(\text{e}\right)=0,\;\left|U_{l}^{t}\right|=0,\;\mu_{l}^{t}\left(\text{e}\right)>0;\\ r_{0j} & \text{if}\;|V_{l}^{t}|>0,\;\alpha_{l}^{t}\left(\text{e}\right)=0,\;\left|U_{l}^{t}\right|=0,\;\mu_{l}^{t}\left(\text{e}\right)=0, \end{array}\right. \end{array}$$ (12)

where the probability *r*_1*j*_ − *r*_0*j*_ is the product of the initial failure 1 − *r*_0*j*_ and the subsequent conditional probability of remission, $\frac{r_{1j}-r_{0j}}{1-r_{0j}}$. Then, for any $\text{e}\in {\left\{0,1\right\}}^{D_{t}}$, the posterior joint probability of donor types at the end of round *t* is

$$\begin{array}{ccc} P_{t}\left(\text{e}\right) & = & P[\text{E}=\text{e}|H_{t}],\\ & = & \frac{1}{Z}\prod_{d=1}^{D_{t}}p^{e_{d}}{\left(1-p\right)}^{1-e_{d}}\prod_{j=1}^{2}\prod_{l\in [N_{j}]}\pi_{l}^{t}\left(\text{e}\right), \end{array}$$ (13)

where *Z* is the normalization constant defined as

$$\begin{array}{c} Z=\sum_{\text{e}\in {\left\{0,1\right\}}^{D_{t}}}\prod_{j=1}^{2}\prod_{l\in [N_{j}]}\pi_{l}^{t}\left(\text{e}\right). \end{array}$$ (14)

Given the posterior joint probability of donors types in Eqs (13) and (14), the posterior marginal probability of each donor *d* being of type 1 can be computed as in Eq (11).

### The Allocation Algorithm

For a given pool size *s* and for a given number of initial donors *D*_1_, we consider the following strategy, which applies to both versions of the model, for assigning patients who are not in remission to donor pools over *T* treatment rounds. Our approach is to allocate donor pools consisting of the donors who are most likely to be of type 1 (i.e., effective). Let $K_{t}=\left(\begin{array}{c} D_{t}\\ s \end{array}\right)$ be the number of pools of size *s* that can be formed from the *D*_*t*_ donors available in round *t*.

**Step 1: Initially Allocate Patients to Donor Pools in a Balanced Manner**: In round *t* = 1, assign $\left\lfloor \frac{N_{1}}{K_{1}}\right\rfloor$ patients of type 1 and $\left\lfloor \frac{N_{2}}{K_{1}}\right\rfloor$ patients of type 2 to each of the *K*_1_ donor pools, where ⌊*x*⌋ is the largest integer less than or equal to *x*. The remaining $N_{1}-\left\lfloor \frac{N_{1}}{K_{1}}\right\rfloor K_{1}$ type 1 patients are assigned to pools $k=1,\ldots ,N_{1}-\left\lfloor \frac{N_{1}}{K_{1}}\right\rfloor K_{1}$, respectively. If $N_{1}-\left\lfloor \frac{N_{1}}{K_{1}}\right\rfloor K_{1}+N_{2}-\left\lfloor \frac{N_{2}}{K_{1}}\right\rfloor K_{1}\leq K_{1}$, then assign the remaining $N_{2}-\left\lfloor \frac{N_{2}}{K_{1}}\right\rfloor K_{1}$ type 2 patients to pools $N_{1}-\left\lfloor \frac{N_{1}}{K_{1}}\right\rfloor K_{1}+1,\ldots ,N_{1}-\left\lfloor \frac{N_{1}}{K_{1}}\right\rfloor K_{1}+N_{2}-\left\lfloor \frac{N_{2}}{K_{1}}\right\rfloor K_{1}$, respectively; otherwise, assign them to pools $N_{1}-\left\lfloor \frac{N_{1}}{K_{1}}\right\rfloor K_{1}+1,\ldots ,K_{1}$ and $1,\ldots ,N_{1}-\left\lfloor \frac{N_{1}}{K_{1}}\right\rfloor K_{1}+N_{2}-\left\lfloor \frac{N_{2}}{K_{1}}\right\rfloor K_{1}-K_{1}$, respectively. Observe the treatment results in round 1 and let *t* = 2.

**Step 2: Compute Posterior Probabilities:** Given the observations at the end of round *t* − 1, compute the posterior marginal probability *ϵ*_*d*_(*t* − 1) for each donor *d* = 1, 2, …, *D*_*t*−1_ using Eqs (8)–(10) for the independence version and Eqs (12)–(14) for the dependence version.

**Step 3: Add Naive Donors:** Before allocating treatment, we consider adding naive (i.e., previously unused) donors. At the beginning of round *t*, identify each donor *d* for which the following happens for the first time: he has treated more than $\bar{n}$ different patients and his posterior marginal probability is below *p* (i.e., *ϵ*_*d*_(*t* − 1) < *p*), where $\bar{n}$ is a user-defined parameter. For each of these donors, add a naive donor to the system, form the new pools (combining the old and new donors) and update the posterior joint probability of each donor’s type at the end of round *t* − 1, which is denoted by *P*_*t*−1_(*e*_1_, …, *e*_*D*_*t*−1__) and derived in Eqs (8)–(10) and (12)–(14) for the two versions of the model, as follows. If *u* = *D*_*t*_ − *D*_*t*−1_ naive donors are added at this step in round *t*, then

$$\begin{array}{c} P_{t-1}\left(e_{1},\ldots ,e_{D_{t-1}},{\hat{e}}_{1},\ldots ,{\hat{e}}_{u}\right)=P_{t-1}\left(e_{1},\ldots ,e_{D_{t-1}}\right)\prod_{d=1}^{u}p^{{\hat{e}}_{d}}{\left(1-p\right)}^{1-{\hat{e}}_{d}}. \end{array}$$ (15)

Also, for the newly added donors *d*, define

$$\begin{array}{c} \epsilon_{d}\left(t-1\right)=p. \end{array}$$ (16)

Furthermore, if there is a patient who has been treated by more than *D*_*t*_ − *s* donors and is not in remission (i.e., this patient does not have access to *s* donors who have not treated him yet), we add as many naive donors as necessary for this patient to have a pool of *s* unexplored donors. After adding these naive donors, all the possible pools are formed and *P*_*t*−1_(**e**) and *ϵ*_*d*_(*t* − 1) are again updated according to Eqs (15) and (16).

**Step 4: Reassign Patients who are not in Remission.** For each patient who is not in remission and among those donors who have not yet treated him, assign the patient to the pool consisting of the *s* donors with the highest values of *ϵ*_*d*_(*t* − 1).

**Step 5: Iterate.** Observe the outcome of the new treatments. Increase *t* by one and if *t* ≤ *T*, return to Step 2. Otherwise, stop.

### Parameter Estimation

Four of the 38 patients in [2] had UC <1 year, and we assume that *N*_1_ = 10 and *N*_2_ = 90 patients (see Table 1 for a list of parameter values). We let *T* = 5 treatment rounds and consider scenarios characterized by various combinations of *D*_1_ = 5, 10 and 20 initial donors, and pool sizes of *s* = 1, 2, 3, 4 and 5. We note that capacity constraints should not be a problem: each UC patient received $\frac{50\text{mL}}{\text{wk}}\times \frac{50\text{g}}{300\text{mL}}=$ 8.33 grams of stool per week in [2], while the average production rate per donor is 87.2 g/day [7], implying that each donor is capable of serving $\frac{7\times 87.2}{8.33}=73.3$ patients in an ongoing manner. In [2], one donor achieved seven remissions out of 18 patients, and the remaining four donors collectively achieved two remissions out of 20 patients. Consequently, we set *p* = 0.2. In addition, three of four type 1 patients and six of 34 type 2 patients experienced remission, although we do not know how many type 1 patients received donations from the most effective donor in [2]. We arbitrarily set *r*_11_ = 0.9, and estimate the remaining three remission probabilities by jointly solving $\frac{4}{38}r_{11}+\frac{34}{38}r_{12}=\frac{7}{18}$, $\frac{1}{5}r_{11}+\frac{4}{5}r_{01}=\frac{3}{4}$, and $\frac{1}{5}r_{12}+\frac{4}{5}r_{02}=\frac{6}{34}$, which yields *r*_12_ = 0.329, *r*_01_ = 0.713 and *r*_02_ = 0.138.

**Table 1:** The model’s parameters, along with their descriptions and values.

Parameter	Description	Value
*N*_1_	number of type 1 (UC < 1 yr) patients	10
*N*_2_	number of type 2 (UC > 1 yr) patients	90
*T*	number of treatment rounds	5
*D*_1_	initial number of fecal donors	5, 10, 20
*p*	proportion of donors that are of type 1 (effective)	0.2
*r*_01_	remission probability for a type 0 donor and type 1 patient	0.713
*r*_02_	remission probability for a type 0 donor and type 2 patient	0.138
*r*_11_	remission probability for a type 1 donor and type 1 patient	0.9
*r*_12_	remission probability for a type 1 donor and type 2 patient	0.329
*s*	pool size	1, 2, 3, 4, 5
$\bar{n}$	minimum sample size before adding a new donor	15

## Results

The proposed algorithm has three user-defined parameters: the threshold $\bar{n}$ for adding naive donors, the pool size *s*, and the number of initial donors *D*_1_. Fixing *D*_1_ = 5, we perform an initial comparison of $\bar{n}=5$, 10 and 15 (Fig A in S1 File), which reveals that the proportion of patients in remission is very insensitive to $\bar{n}$ in the independence and dependence versions of the model. Consequently, we use $\bar{n}=15$ throughout the study. For most combinations of pool size *s* = 1, …, 5 and number of initial donors *D*_1_ = 5, 10 and 20 and for the independence and dependence versions of the model (some combinations of high values of (*s*, *D*_1_) were computationally intractable, particularly for the dependence version), we simulate *T* = 5 rounds of treatment 200 times and plot *R*_*t*_ vs. *t*, where *R*_*t*_ is the mean (over the 200 realizations) proportion of patients (of both types) in remission at the end of round *t*, for *t* = 1, …, 5. We also compare the performance of our proposed algorithm to that of a random policy, which does not add any naive donors and randomly reassigns each patient who is not in remission to one of the existing donors who has not previously treated them. In the independence version of the model, this policy has a remission proportion at the end of round *t* = 1, …, *D*_1_ of

$$\begin{array}{c} \frac{\sum_{j=1}^{2}\sum_{\tau =1}^{t}N_{j}\left[pr_{1j}+\left(1-p\right)r_{0j}\right]{\left(1-\left[pr_{1j}+\left(1-p\right)r_{0j}\right]\right)}^{\tau -1}}{\sum_{j=1}^{2}N_{j}}, \end{array}$$ (17)

and no additional patients achieve remission after round *D*_1_. There is no simple analogous expression for this quantity in the dependence version.

### Independence Version of the Model

**Fig 2:**
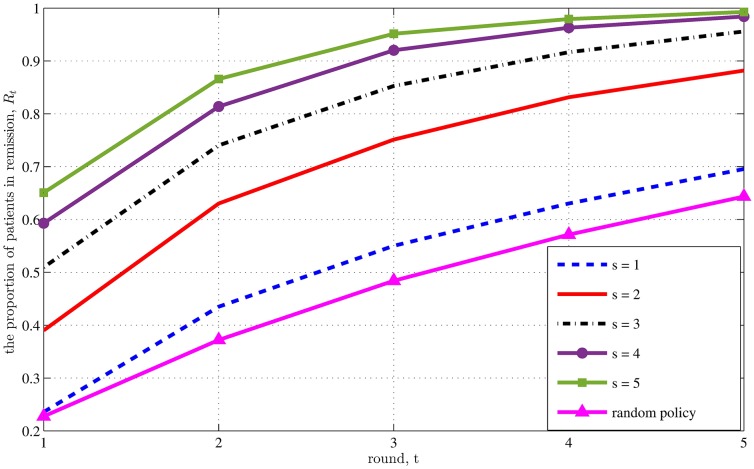
Results for the independence version of the problem with *D*_1_ = 5 initial donors.

We know from Eq (1) that *R*_*t*_ converges to 1.0 as the pool size *s* or the treatment round *t* approaches infinity. Starting with the case where *D*_1_ = 5 initial donors (Fig 2), we see that convergence is somewhat rapid: by the end of round 5, over 95% of patients are in remission when *s* ≥ 3 (Fig 2). Although increasing the pool size achieves decreasing returns, there is a large improvement in remission from increasing the pool size from *s* = 1 to *s* = 2 for each of the first five rounds; e.g., at the end of rounds 2 and 3, approximately 50% more patients are in remission when *s* = 2 compared to when *s* = 1. Indeed, the impact from increasing the pool size *s* from 1 to 2 is much larger than the impact from switching from the random policy to the *s* = 1 policy.

When the initial number of donors is increased from five to 10, the results are qualitatively identical, although the remission probabilities are slightly higher (Fig Ba in S1 File). When the initial number of donors is increased to 20, the remission probability increases relative to the *D*_1_ = 10 cases when there is no pooling (i.e., *s* = 1), but is very similar to the *D*_1_ = 10 cases when there is pooling (Fig Bb in S1 File). Returning to the base case where *D*_1_ = 5, we also note that the number of donors in round *t*, *D*_*t*_, increases with the pool size *s*, although the dependence does not have a strong effect until round *t* = 3 (Fig Ca in S1 File). The number of donors is nearly linear (i.e., *D*_*t*_ = *D*_1_ + *s*(*t* − 1)) for *s* = 4 and 5. For this set of parameter values, the addition of naive donors during step 3 of the algorithm was primarily caused by patients exhausting all available donors, not because donors were deemed ineffective.

### Dependence Version of the Model

**Fig 3:**
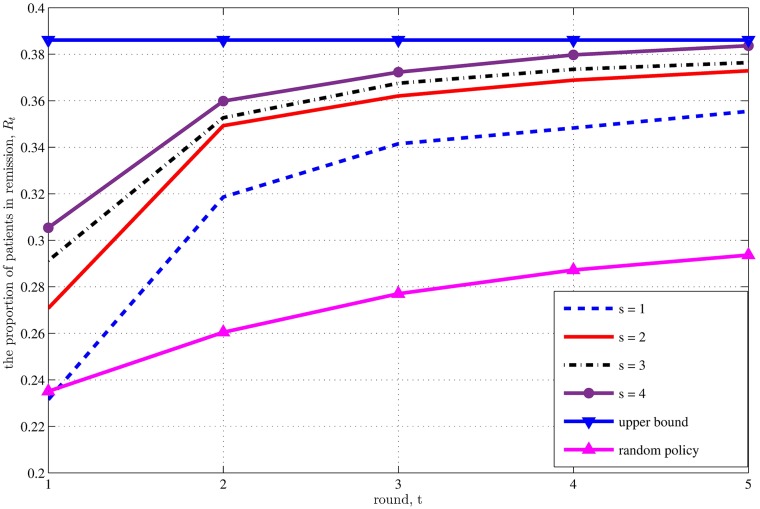
Results for the dependence version of the problem with *D*_1_ = 5 initial donors.

In the dependence version of the model, the maximum remission probability for a type *j* patient is *r*_1*j*_, and hence *R*_*t*_ converges to

$$\begin{array}{c} \frac{N_{1}r_{11}+N_{2}r_{12}}{N_{1}+N_{2}}=0.3861 \end{array}$$ (18)

as *s* → ∞ or *t* → ∞, which is the remission proportion if everyone is eventually treated by a pool with an effective donor. As in the independence version of the model, the remission proportion approaches the asymptotic limit at the end of round 5 for *s* ≥ 3 (Fig 3). As expected, the dependence version achieves much lower remission proportions than the independence version of the model, and few remissions occur after the second round of treatment. The impact of pooling is more modest in this version of the problem, both in absolute and relative increases in *R*_*t*_. In contrast to the independence version, here the improvement from switching from the random policy to the *s* = 1 policy is much larger than the improvement from switching from *s* = 1 to *s* = 2. Nonetheless, improvements from pooling are still meaningful: e.g., at the end of round 2, 15% more patients are in remission if the pool size is increased from *s* = 1 to *s* = 5. Unlike in Fig 2, the impact of increasing the pool size does not consistently exhibit decreasing returns in Fig 3. As in the independence version of the model, we see that increasing the number of initial donors from five to 10 gives qualitatively similar results, but with slightly higher remission probabilities (Fig D in S1 File). The number of donors in round *t*, *D*_*t*_, is nearly identical in the independence and dependence versions of the model when *D*_1_ = 5 (Fig Cb in S1 File).

### Sensitivity Analysis

The parameter with the most uncertain value is *p*, which is the proportion of donors who are effective. This value may vary for different chronic microbiota-associated diseases, and may increase over time as methods are developed to identify effective donors *a priori* [8]. Fixing *D*_1_ = 5 initial donors and changing *p* from the base-case value of 0.2 to 0.1 and 0.5, we find that, as expected, the remission probability increases with *p* (Fig E in S1 File). The results are qualitatively similar to the base case of *p* = 0.2, except that with no pooling (i.e., *s* = 1) in the *p* = 0.1 case, the performance of the proposed algorithm is very similar to the performance of the random policy, perhaps because of the small expected number of effective initial donors.

Because there is also uncertainty about whether either of the two versions of the model is accurate, we assess the robustness of our algorithm by supposing that the dependence model is the true model of the system, but that we incorrectly believe that the independence model is the true model. That is, the algorithm uses the posterior probabilities based on the independence version of the model, but we simulate the performance of this algorithm using the true dependence version of the model, and compare it to the performance under the dependence version of the model (i.e., where the algorithm correctly uses the posterior probabilities based on the dependence version). For *D*_1_ = 5 initial donors and pool sizes of *s* = 1, 2, 3, we see that the algorithm performs reasonably well even when it assumes the wrong version of the model (Fig F in S1 File).

## Discussion

FMT for treatment of chronic microbiota-associated diseases such as UC is in its infancy. The very little clinical data that have been generated suggest considerable donor heterogeneity in treatment outcomes [2]. While some of this heterogeneity may eventually be explained using 16s rRNA data or metagenomic analysis, it is caused by donor characteristics that are currently unobservable. Unobservable donor heterogeneity and the chronic nature of the disease (and hence the ongoing nature of treatment) suggest that treatment outcomes can be improved by (i) using an adaptive design in multi-round treatment settings, where past treatment results are used to infer donor efficacy and to reassign patients who are not in remission to promising donors, and (ii) pooling stools, which generates a form of combination therapy, as is commonly used to treat cancer, HIV and psychiatric disorders. Here, we use mathematical modeling to assess the potential of approaches (i) and (ii) to improve treatment outcomes.

Our results suggest that modest (e.g., pool size *s* = 2 or 3) pooling of stools can increase the proportion of patients who achieve remission, particularly in the first few rounds of treatment, although the improvements are smaller under the dependence version of the model than the independence version. These conclusions are robust to the number of initial donors (more specifically, the ratio of the number of patients to the number of initial donors) and the likelihood that each donor is effective. In addition, even with no pooling, performance can be improved by continually updating the likelihood that each donor is effective and adding naive donors when existing donors appear to be ineffective or when patients have explored all current donors.

Our two versions of the model consider two extreme sets of probabilistic assumptions: independence of treatment results (among different donors in a pool, and across different treatment rounds) and perfect dependence. Although the truth is likely to reside in between these two extremes, these two versions are the natural starting points in the absence of further information. Nonetheless, the fact that pooling provides improvements in both versions of the model suggests that pooling should also be beneficial in the more realistic setting of partially-correlated treatment results. Moreover, our sensitivity analysis, where the algorithm incorrectly assumes the independence version of the model and yet still performs well when the ground truth is the dependence version of the model, suggests that our algorithm is very robust with respect to the underlying probabilistic assumptions in the model.

Pooling aside, the difference in remission proportions between the two versions of the model is very large. Because the independence and dependence versions of the model can be mapped into a setting with many or one, respectively, effective and ineffective factors (e.g., microorganisms), it is possible to develop intermediate models with several different effective and ineffective factors; alternatively, we could use the multivariate Bernoulli distribution [9] to develop intermediate versions of the model. Our hope is to fit a family of such models to future data from multi-round clinical trials in order to assess the amount of positive correlation that exists, thereby allowing us to both improve our understanding of multi-round FMT treatment for microbiota-related diseases and refine our predictions and recommendations.

Over the coming years, analyses of 16s rRNA data from donors and patients (or more general metagenomic analysis) may shed light on the underlying factors that determine remission [8]. This new information should allow the concept of optimal pooling to become more refined, by combining stools from donors whose microbiota are complementary to one another and to the patient.

The pooling assumption—in particular, that daily cycling of pills from *s* patients achieves the same clinical outcome as when the stools from *s* donors are actually pooled—breaks down for some value of *s*, although this limit has yet to be evaluated empirically. Fortunately, much of the improvement from pooling can be achieved with pool sizes no larger than *s* = 3 (Figs 1 and 2), and so the practical benefits of pooling can be realized even if the pooling assumption breaks down at moderate levels of *s*.

We should also note that—to the extent that FMT may be risky—e.g., due to the presence of untested agents in stools or to the theoretical risk of becoming more susceptible to chronic conditions such as obesity or autoimmune disorders [10]—the use of pooled stools increases the number of donors—and thereby the risk—that each patient is exposed to. Moreover, if there was an adverse event while pooling was in use, tracing back to a particular donor would be more costly and difficult.

We conclude with two technical points. In the absence of pooling, it would be straightforward to take a Bayesian approach to this problem—i.e., assuming that we have prior distributions of the *r*_*ij*_ values that are updated throughout the rounds of treatment—using the beta-Bernoulli conjugate pair [11]. However, this approach appears to break down when the pool size *s* > 1, and a Bayesian approach in the presence of pooling remains a topic for future work.

In addition, our proposed algorithm is not optimal. We also developed a more complicated algorithm that uses the joint probability of donor types in Eqs (8)–(10) and (12)–(14) rather than the marginal probabilities in Eq (11). Exploratory computational analysis (not shown) suggests that the difference in performance between the two policies is negligible for *s* = 1, 2, 3 (we were unable to get the more complicated algorithm to run in a timely manner for *s* > 3).

## Conclusion

We use two extreme probabilistic models—one based on statistical independence of treatment outcomes and the other based on perfect dependence—to assess the efficacy of pooled stools for FMT in the setting of multiple treatment rounds, where patients are adaptively reassigned to donor pools in each round. We predict that pools of size two or three can increase the proportion of patients in remission during the first few rounds of treatment, with the improvements being larger in the independence version of the model. Because pooling is beneficial at both probabilistic extremes, it is likely to be a robust strategy in practice. However, the remission proportion is much different in the two versions of the model, and this uncertainty cannot be resolved without further clinical data. The two versions of our model map into a setting where there are many factors (e.g., microorganism) or one factor that causes remission, and hence we have developed a framework for a family of models with any number of factors. To gain a better understanding of FMT for chronic diseases, data from future multi-round trials should be fit to a family of these models.

## Supporting Information

S1 File Supporting Material. Contains additional figures discussed above.
(PDF)
