# Supplementary material for: Exploring the Efficacy of Pooled Stools in Fecal Microbiota Transplantation for Microbiota-Associated Chronic Diseases
Source: PLoS One. 2017 Jan 9;12(1):e0163956. doi: 10.1371/journal.pone.0163956 (PMC5221766; doi:10.1371/journal.pone.0163956)
Supplement: S1 File — Contains additional figures discussed above. (PDF) [file pone.0163956.s001.pdf]

Supporting Information

Figs A-F are discussed in the main text.

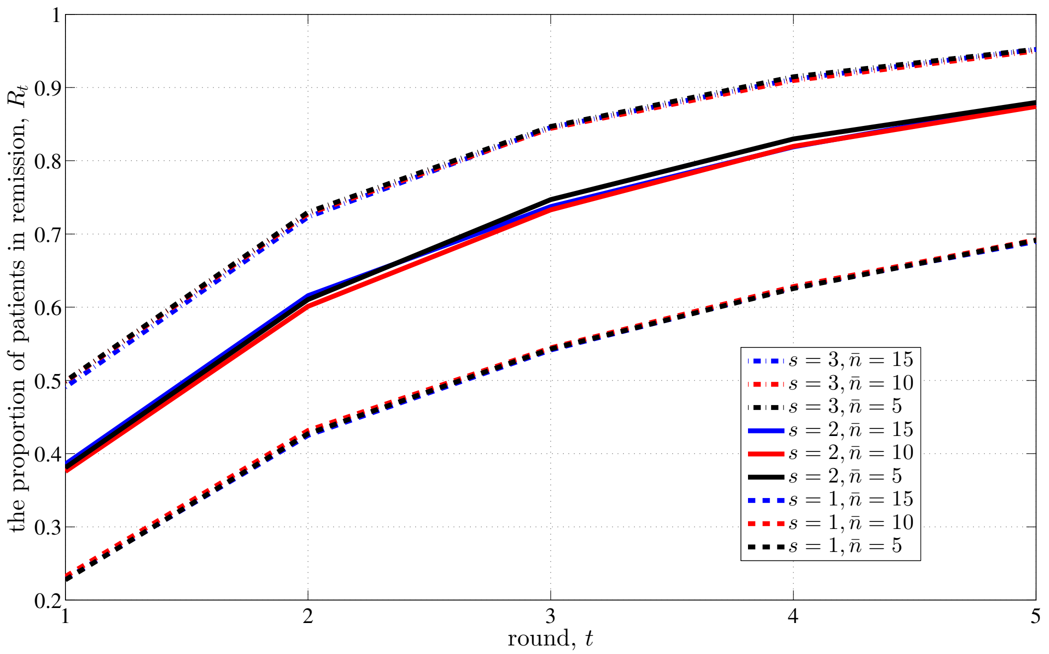

(a)

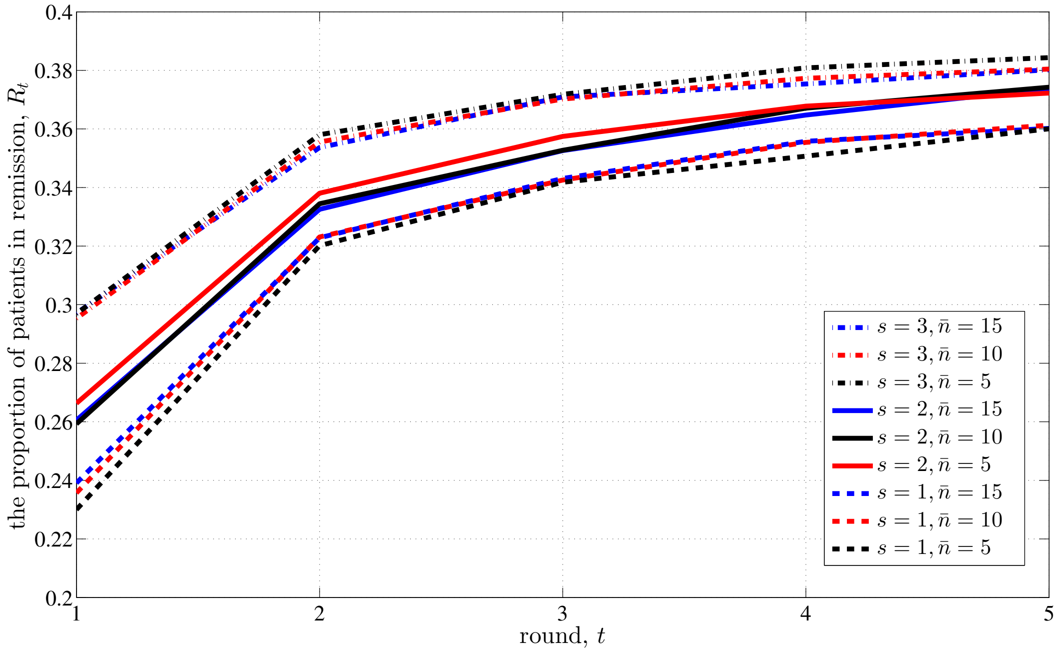

(b)

Fig A: For **(a)** the independence version and **(b)** the dependence version of the model with $D_1 = 5$ initial donors and pool sizes of $s = 1, 2, 3$, the impact of different values of the threshold $\bar{n}$, which is used to add naive donors in step 3 of the algorithm.

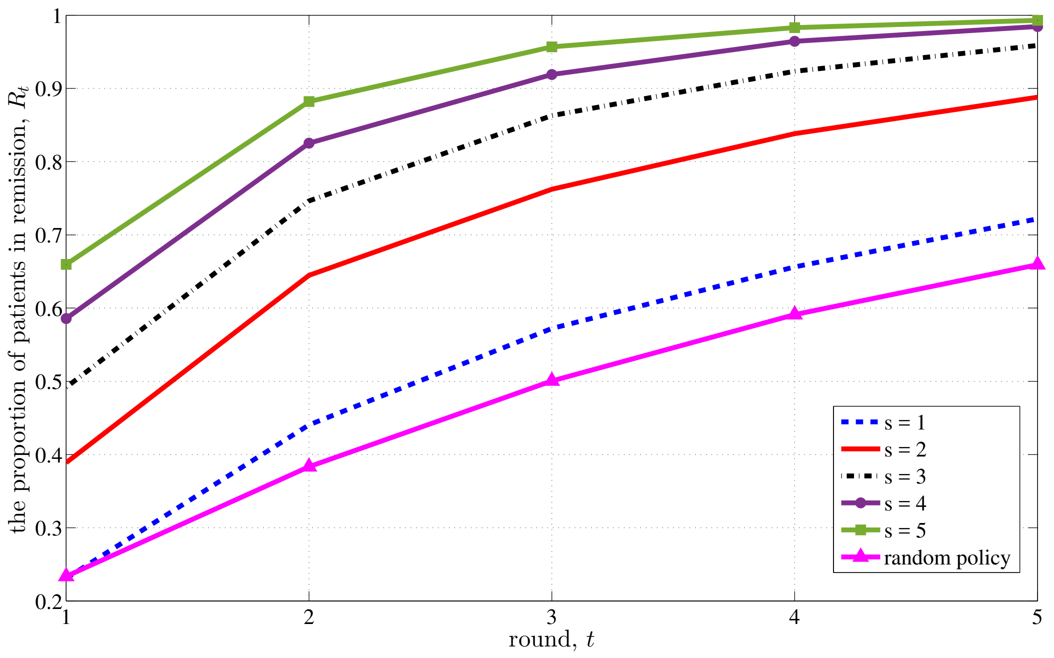

(a)

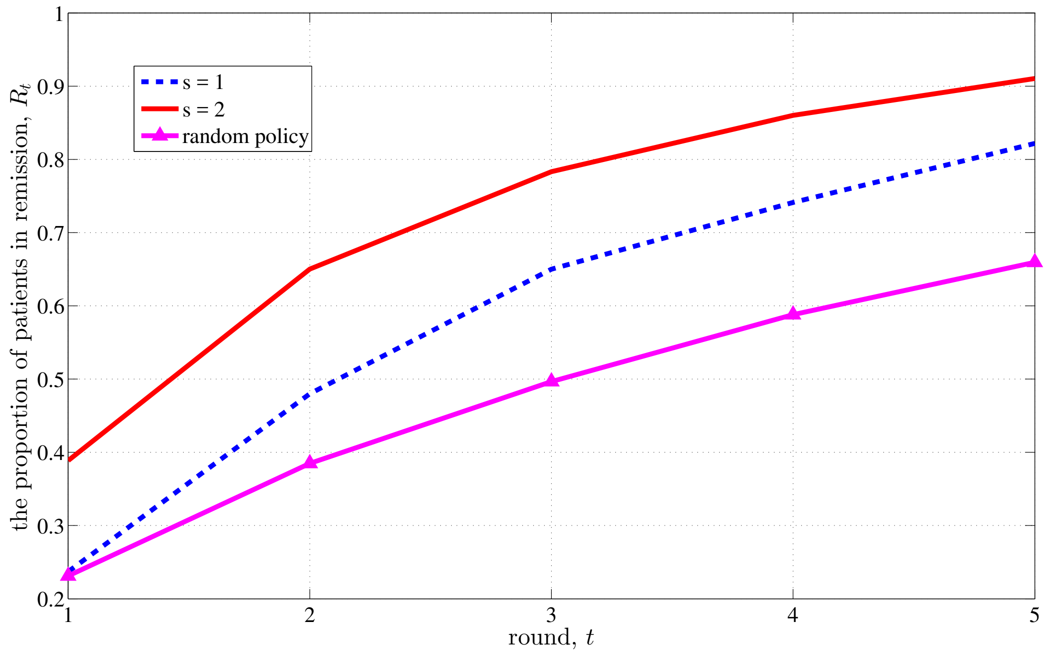

(b)

Fig B: Results for the independence version of the model with **(a)** $D_1 = 10$ and **(b)** $D_1 = 20$ initial donors.

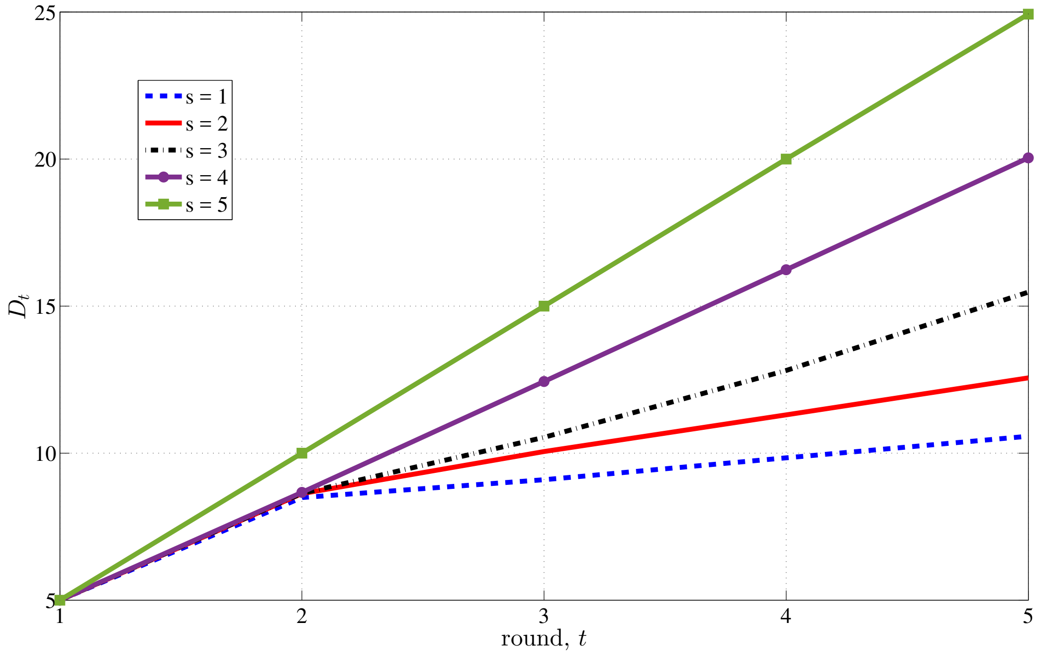

(a)

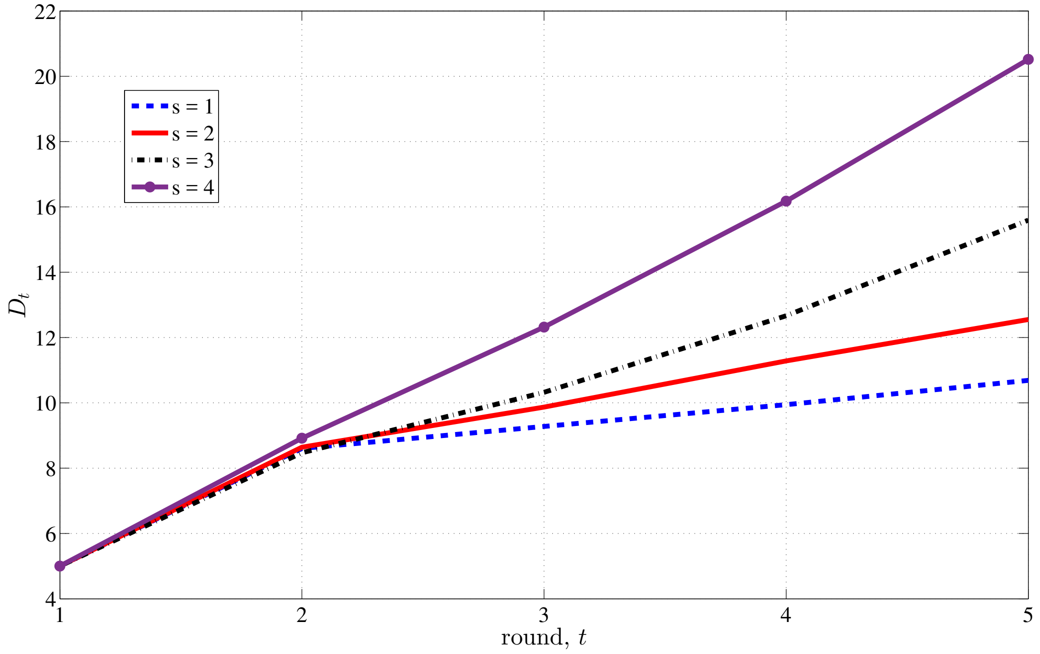

(b)

Fig C: For **(a)** the independence version and **(b)** the dependence version of the model with $D_1 = 5$ initial donors, the total number of donors available in round t, D_t.

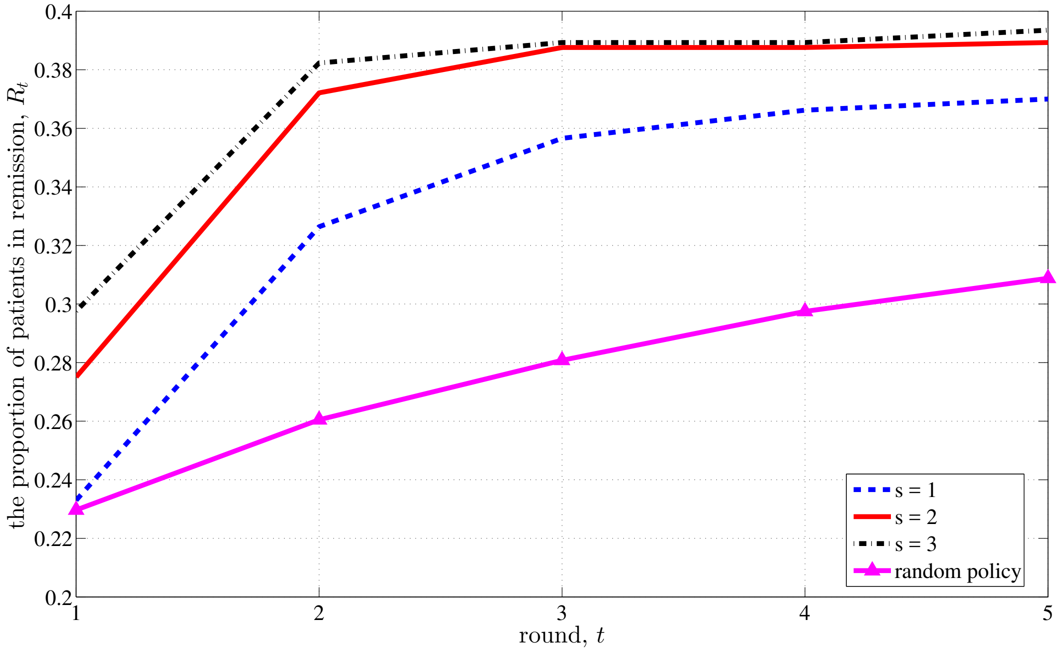

Fig D: Results for the dependence version of the problem with $D_1 = 10$ initial donors.

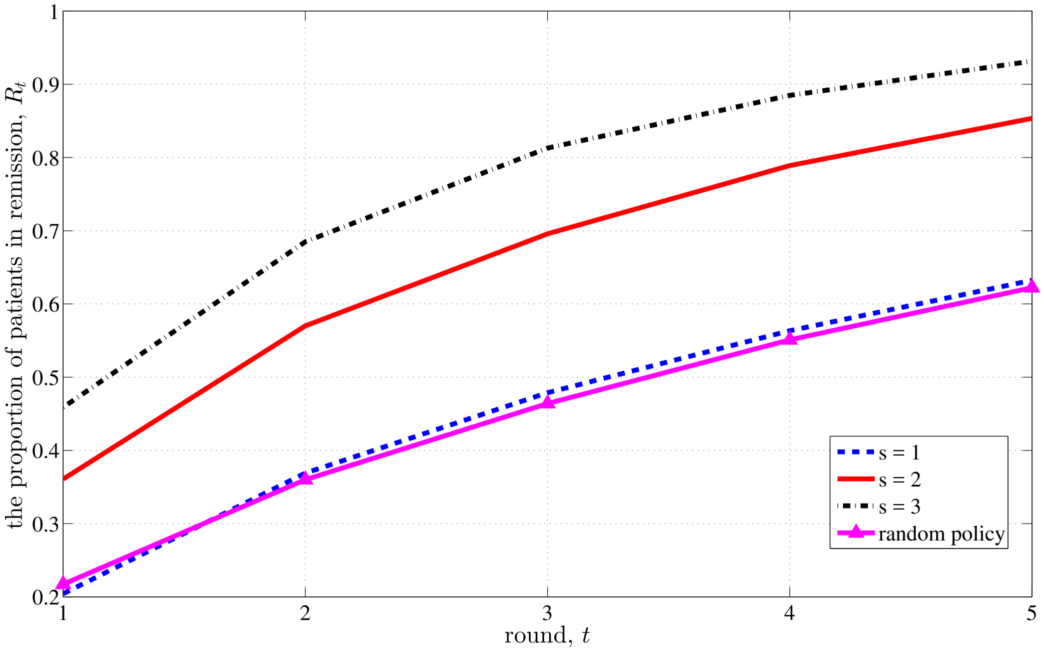

(a)

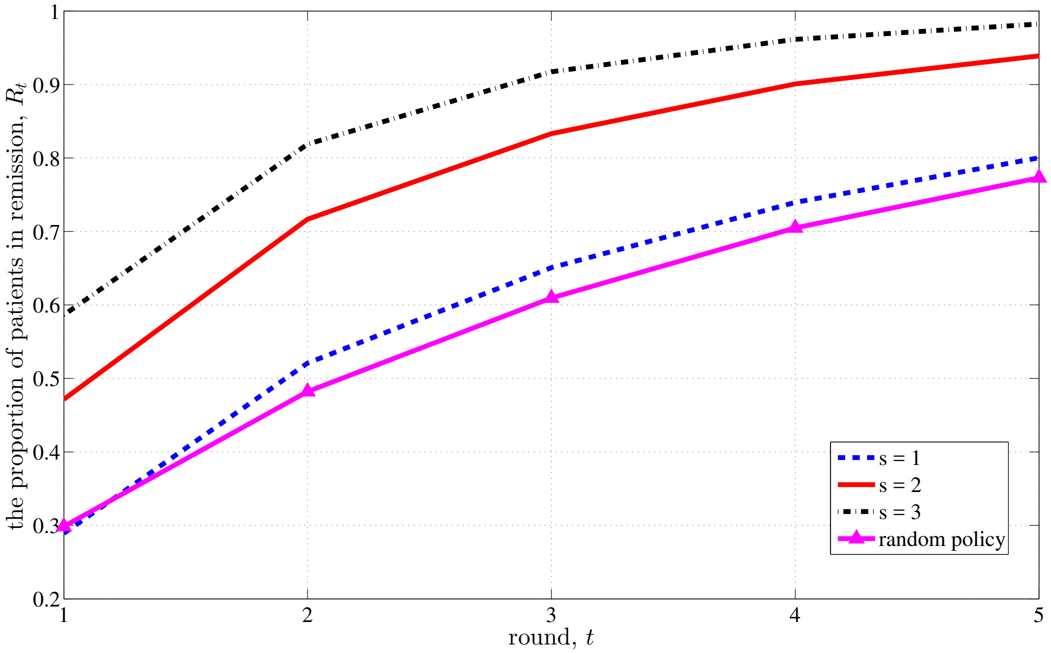

(b)

Fig E: Sensitivity analysis with respect to p, which is the proportion of donors who are effective. Results for the independence version of the model with $D_1 = 5$ initial donors when **(a)** $p = 0.1$ and **(b)** $p = 0.5$.

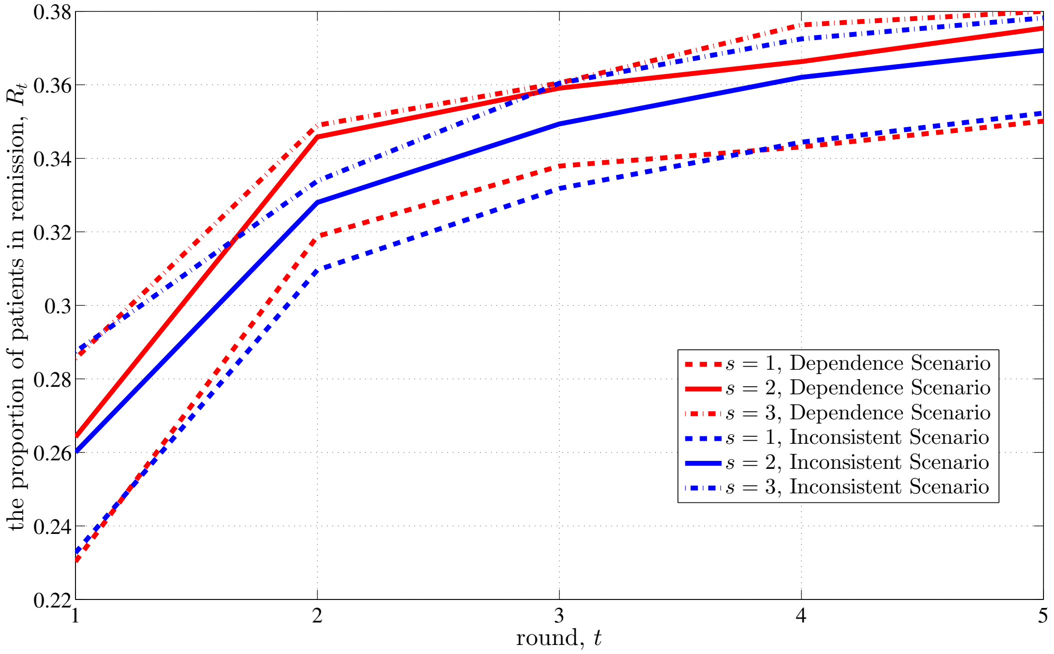

Fig F: Results from a sensitivity analysis, where – in the inconsistent scenario – the algorithm assumes the independence version of the model but the true model is the dependence version.
